# A New Approach for the Microencapsulation of Clitoria Ternatea Petal Extracts by a High-Pressure Processing Method

**DOI:** 10.3390/pharmaceutics13010023

**Published:** 2020-12-24

**Authors:** Hua-Wei Chen, Yu-Wei Chang, Wu-Po Fang

**Affiliations:** Department of Chemical and Materials Engineering, National Ilan University, 1, Sec. 1, Shen-Lung Road, Yilan 260, Taiwan; superlemi@gmail.com (Y.-W.C.); d9006113@gmail.com (W.-P.F.)

**Keywords:** *Clitoria ternatea*, high-pressure processing, microencapsulation, liposome, ultrasonication

## Abstract

Toxic organic solvent residues and the active substances of thermal degradation (such as anthocyanin and polyphenols) are always a concern with the liposomes produced by traditional techniques. The present study focuses on a new approach for the microencapsulation of *Clitoria ternatea* petal (CTP) extracts, which contain anthocyanins, by high-pressure processing (HPP) at room temperature. Thus, a series of CTP liposomes were prepared and their physicochemical properties were analyzed by laser granulometry and by scanning electron microscopy (SEM). The results revealed that the average particle size of the liposomes after HPP treatment increased gradually from 300 MPa to 600 MPa, possibly due to the aggregation of liposomes and damage to the phospholipid bilayers. For the preparation of liposomes by the HPP method at 300 MPa, the mean particle size, polydispersity index (PDI), and encapsulation efficiency were 240.7 nm, 0.37, and 77.8%, respectively. The HPP method provided a number of advantages over conventional methods (magnet stirring and ultrasonication) as it could allow liposome preparation with higher encapsulation efficiency, smaller size, and narrower, more reproducible particle size distribution. Conclusively, microencapsulation in the liposomes was successfully achieved with the fast-adiabatic expansion of HPP.

## 1. Introduction

Recent studies have demonstrated that *Clitoria ternatea* can play a variety of roles: as an antioxidant [1,2], antibacterial agent [3], in scavenging free radicals [4,5], and preventing cardiovascular diseases [2,5]. Phenolic compounds in *Clitoria ternatea* are a diverse group that includes anthocyanins, flavonol glycosides, and proanthocyanidins [1,2,3,4]. However, Anthocyanin as a potential source for antimicrobial activity in the flower petal of *Clitoria ternatea* is among the flavonoids that are the most susceptible to pH levels and temperature [6,7]. Thus, the active substances of thermal degradation (such as anthocyanin and polyphenol) are always a concern with the liposomes produced by traditional techniques.

Microencapsulation is considered to be a means of improving delivery systems that prolong or control drug release and improve targeting of bioactive compounds, bioavailability, and stability [8,9]. Microencapsulation of liposomes, micelles, and polymeric or inorganic nanoparticles has been developed to provide higher therapeutic efficacy, lower toxicity, and controlled delivery of chemotherapeutic agents [9]. The liposomes, which are composed of a phospholipid bilayer, are defined as spherical vesicles with particle sizes ranging from 30 nm to several micrometers [8]. All methods of preparing liposomes typically have the following four major stages: drying down lipids from an organic solvent, dispersing the lipid in aqueous media, purifying the resultant liposome, and analyzing the final product [10]. Additionally, numerous techniques for liposome preparation and size reduction remain popular, such as the French press, sonication, detergent dialysis, reverse-phase evaporation, ethanol injection, homogenization, membrane extrusion, and supercritical fluid process [8,10,11]. However, many techniques have been employed in lab-scale liposome preparation and drug loading, but only a few large-scale techniques (ethanol injection and supercritical fluid process) are available. High-pressure processing (HPP) was officially recognized by the U.S. Food and Drug Administration in 2000 and is a non-thermal pasteurization technology that uses hydrostatic pressure rather than the conventional method of heat to destroy microorganisms in the food industry [12]. Laboratory HPP equipment with different capacities from 0.3 to 10 L have been developed by the supplier (Avure, Stansted Fluid Power, Baotou, Kobelco, and Toyo Koatsu).

The main principle of HPP is as follows: products that have been hermetically sealed are placed in a thermally insulated airtight vessel and subjected to high pressure (commercially at 600 MPa) transmitted by a hydraulic fluid (normally water), which provides a pasteurization effect through the uniform and instantaneous application of a high pressure [13]. Two general scientific principles, namely, Le Chatelier’s principle and the isostatic principle, govern the uniform application of pressure on products in a sealed vessel [13,14]. The application of HPP technology on the preservation of food can damage membranes and denature enzymes but protect the functional ingredients such as polyphenols, protein, colostrum, flavonoids, and probiotics [14,15].

HPP technology preserves food against harmful microorganisms, denature enzymes, and causes changes in cell morphology [14,15].

However, HPP can not only damage the cell membranes of microorganisms but also denature enzymes [15].

The increase of pressure in the cell environment disrupts membrane permeability, which is followed by the loss of membrane integrity and swelling, and eventually leads to cell death.

Compared with traditional thermal processing technology, the food is in packaged form and does not directly contact HPP devices, preventing the secondary contamination of food after pasteurization. Furthermore, HPP is performed at room temperature to reduce energy consumption, and the pressure transfer medium can be recycled and reused after processing. Conclusively, HPP technology is an environmentally friendly processing technology with the advantages of low energy consumption and low contamination risk [16].

As mentioned previously, the thermal degradation of anthocyanins and polyphenol has been extensively studied; however, there are few studies on the prevention of thermal degradation of anthocyanins and polyphenol [17], and very few pieces of literature are related to the application of HPP on liposome preparation. Thus, the objective of this study was to encapsulate *Clitoria ternatea* petal (CTP) extracts in liposomes using a newly designed combination of ethanol injection and HPP to improve the encapsulation efficiency, mean particle size, and size distribution of liposomes, as well as avoid the addition of toxic organic solvents and the thermal degradation of active substances.

## 2. Material and Methods

### 2.1. Materials and Preparation of Clitoria Ternatea Petal Extract

The dry *Clitoria ternatea* petal (Toucheng farm), Folin–Ciocalteu reagent (Scharlau, Barcelona, Spian), Gallic acid (Acros Organics, Bridgewater, NJ, USA), L-alpha-Lecithin (Acros Organics, Bridgewater, NJ, USA), Cholesterol (Acros Organics, Bridgewater, NJ, USA), Ethanol absolute (Thermo Fisher Scientific Inc. (Waltham, MA, USA), and Phosphotungstic acid (Scharlau, Barcelona, Spian) used for the preparation of stock solutions were reagent grade and were used without further purification. To start, 5 g of dry *Clitoria ternatea* petal was ground and added to 100 mL of distilled water, which was then heated and stirred at 50 ℃ for two hours. The solution was filtered through filter paper and freeze-dried to obtain *Clitoria ternatea* petal (CTP) extracts. The freeze-dried CTP extracts were stored at 4 °C for further application.

### 2.2. Preparation of Liposomes

**Figure 1:**
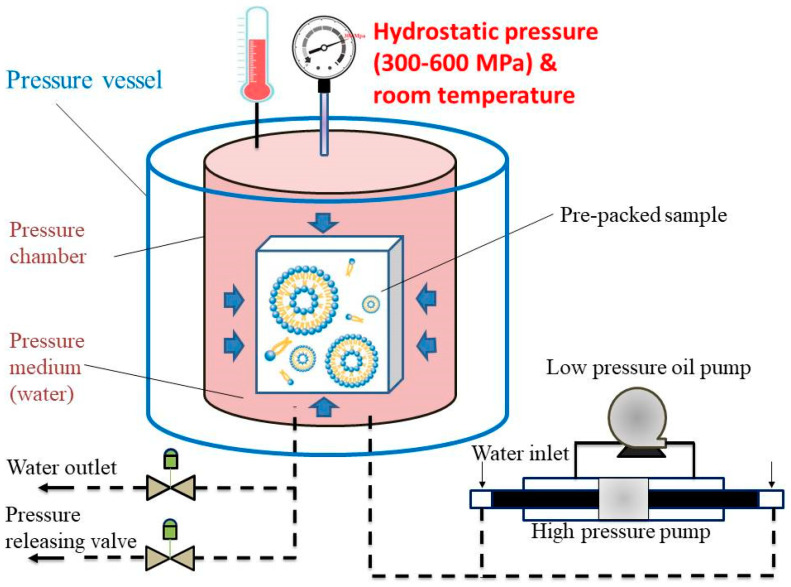
The schematic diagram of the high-pressure processing (HPP).

Appropriate amounts of lecithin and cholesterol were first dissolved by stirring in absolute ethanol for the preparation of liposomes by the ethanol injection method. The lipid solution (1:0.15 ratio of L-alpha-Lecithin to cholesterol) was injected into the desired volume of 0.7% CTP extracts using a syringe and then homogenized with stirring, ultrasonic oscillation, or the HPP method. After the organic phase interacted with the aqueous phase, liposomes were formed by HPP (HPP600MPa/6.2L, BaoTou KeFA High-pressure Technology Co., Ltd., Baotou Kefa, China) in a 6.2 L vessel using water as the pressure-transmitting medium within 10 min at 25 °C (Figure 1). The pre-packed samples (1:0.15 ratio of L-alpha-Lecithin to cholesterol) in flexible bags (linear low-density polyethylene, LLDPE) were loaded into the HPP chamber. HPP must be done in flexible packaging; rigid packages would not be able to transmit pressure internally. In pascalization, samples in flexible bags are sealed and placed into a steel chamber containing water, and pumps are used to create isostatic pressure. The pumps may apply pressure constantly or intermittently. The sample was held at an isostatic pressure up to the appropriate pressure (200–600 MPa) for liposome preparation. Lastly, the HPP product (liposome) came out of the vessel and is ready. The effects of pressure on the liposomes were examined under different pressures (300, 400, 500, and 600 MPa).

### 2.3. Characterization of Liposomes

The image of liposomes and the particle sizes were analyzed by a transmission electron microscopy (TEM, HITACHI HT7700, Hitachi High-Tech Corporation, Tokyo, Japan) at 75 kV and a dynamic light scattering (DLS, Zetasizer Nano S90, Malvern Instruments Ltd, Worcestershire, UK) with a refractive index of 1.33, respectively. Furthermore, the cumulative analysis from DLS is typically the mean value from the intensity distribution and the polydispersity index (PDI) to describe the width of the assumed Gaussian distribution.

The quantification of the total phenolic content (TPC) was performed by a UV/VIS spectrophotometer (INESA, 752N, Inesa Analytical Instrument Co., Ltd., Shanghai, China) at 760 nm according to a previously-described study [18]. The encapsulation efficiency was calculated according to the equation below:

(1) $$\mathrm{Encapsulation}\;\mathrm{efficiency}\;\left(\%\right)=\frac{TPC_{e}}{TPC_{i}}\times 100\%$$

where *TPC_e_* is the total phenol content encapsulated in the beads and *TPC_i_* is the total phenol content in the initial extract solution used for the encapsulation process.

## 3. Results and Discussion

### 3.1. Liposome Morphology

**Figure 2:**
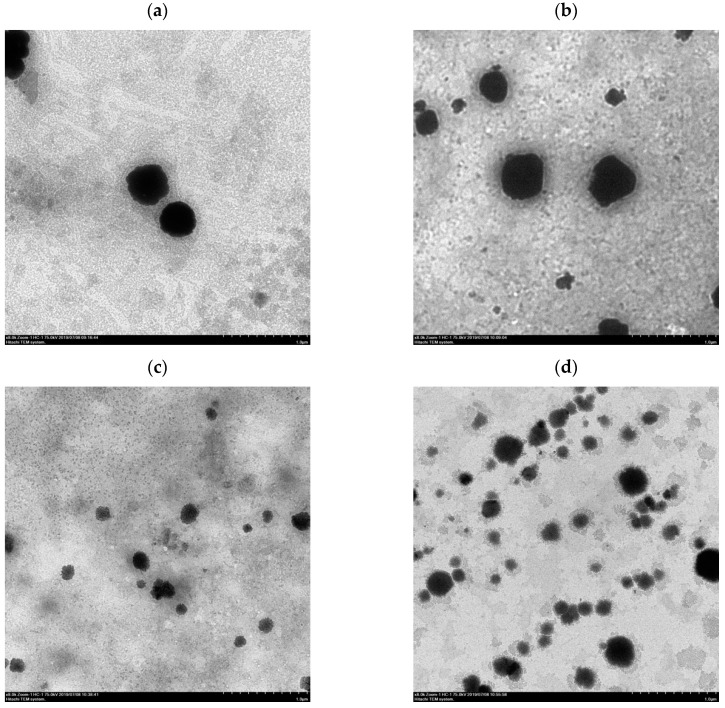

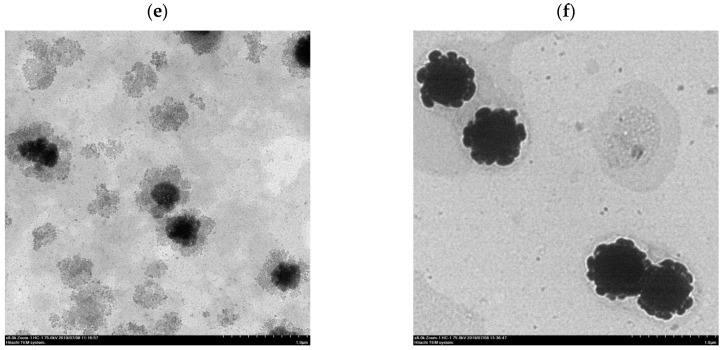
Transmission electron microscopy (TEM) images of *Clitoria ternatea* petal (CTP) liposomes prepared by different homogenization types. (**a**) Magnet stirring; (**b**) Ultrasonic bath; (**c**) Probe ultrasonication; (**d**) HPP at 300 MPa; (**e**) HPP at 400 MPa; (**f**) HPP at 600 MPa.

After the ethanol injection method, the liposomes were destroyed by homogenization through stirring, ultrasonic oscillation, or HPP to form a dispersed suspension of phospholipid fragments that were then reassembled to form liposomes. The morphology of the liposomes prepared according to different homogenization methods is shown in Figure 2. TEM images (Figure 2c,d) revealed that the particle size of the liposomes by probe ultrasonication and HPP at 300 MPa was from 100 to 200 μm and from 150 to 300 μm, respectively. The lipid fragments and lipid granules were formed under different homogenization methods, and the particle size of the liposomes followed the order of magnet stirring > ultrasonic bath > HPP at 300 MPa > probe ultrasonication.

### 3.2. Liposomes of CT Petal Extract Prepared by the HPP Method

#### 3.2.1. Effect of Pressure on Liposome Formation

**Figure 3:**
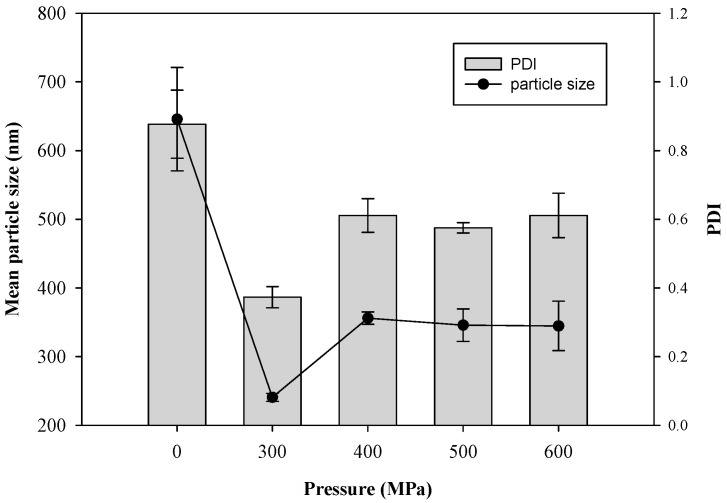
Effect of pressure on mean diameter and polydispersity index (PDI) of CTP liposomes prepared by the HPP method.

Pressure is an important processing parameter in HPP. Thus, the effects of hydrostatic pressure (from 300 MPa to 600 MPa) on the particle size of CTP liposomes by the HPP treatment were investigated, as shown in Figure 3. The mean particle size of liposomes (blank) without HPP treatment (0 MPa) was 645.8 ± 75.2 nm. The results demonstrated that the liposomes resulting from the HPP treatment at 300 MPa by a pressurization and depressurization process had a significantly decreased mean particle size compared to those created without the HPP treatment because of the dispersion and destruction effect via the fast-adiabatic expansion of water. The double-layered phospholipids were compressed and packed tightly by the hydrostatic pressure during pressurization processing. During decompression, the structure of the phospholipid bilayer was lost and transient phospholipid fragments were formed due to the fast-adiabatic expansion of water. Lastly, the transient phospholipid fragments self-assembled rapidly in solutions to form liposomes with smaller particle sizes.

After HPP treatment, the average particle size of the liposomes increased gradually from 240.7 ± 5.8 nm to 344.6 ± 36.0 nm with the increase of pressure from 300 MPa to 600 MPa, possibly owing to the aggregation of liposomes and damage of the phospholipid bilayers. The results revealed that excessive compression possibly caused the destruction of the phospholipid bilayers by HPP at 600 MPa of pressure, which also was in accord with the aggregation of liposomes imaged by TEM (Figure 2f). Hydrostatic pressures on critical thresholds may reflect that cell membrane systems, i.e., phospholipid bilayers and intrinsic transport proteins, are drastically and irreversibly damaged [19].

#### 3.2.2. Particle Size Distribution

**Figure 4:**
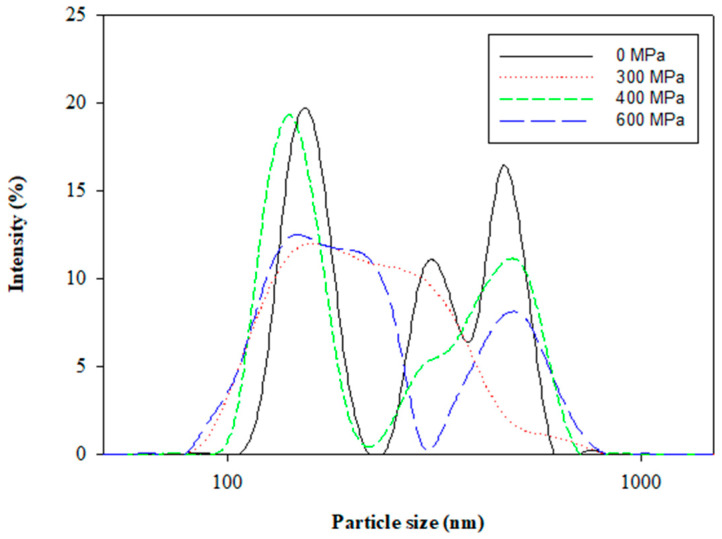
Size distribution of *Clitoria ternatea* petal extract-loaded liposomes prepared at different pressures by the HPP method.

The polydispersity index (PDI) indicates the size uniformity of a liposome, which is related to the stability of the colloidal system. The effects of pressures from 300 MPa to 600 MPa by HPP on the polydispersity PDI and the size distribution curve of the liposomes are shown in Figure 2 and Figure 3, respectively. The PDI (0.37 ± 0.03) with the HPP treatment at 300 MPa was smaller than that (0.88 ± 0.10) without the HPP treatment. The results of the average particle size and PDI in this study revealed that the HPP treatment could reduce the particle size and enhance the uniformity of the liposomes. The PDI, as well as the average particle size, showed a similar trend from 300 MPa to 600 MPa. The results inferred that excessive compression possibly caused the aggregation of liposomes and the bimodal size distribution (Figure 4) as a certain pressure was exceeded. The previous research concluded that the successive compression above a certain pressure caused the aggregation of surfactants in aqueous solutions [20]. In a previous study of PEG-induced fusion, osmotic mismatches have been used to enhance aggregation, as negative osmotic pressure (when the solution inside liposomes is hypotonic compared to the solution around liposomes, normally associated with shrinkage of liposomes) promoted aggregation of LUVs in the presence of PEG [21]. From the size distribution curve (Figure 4), it was found that the liposomes prepared by the HPP method under 300 MPa of pressure had a left-shifted particle size distribution, which reduced the mean particle size. The results also referred to the display of the unimodal distribution as a whole in drug delivery systems.

#### 3.2.3. Encapsulation Efficiency

**Figure 5:**
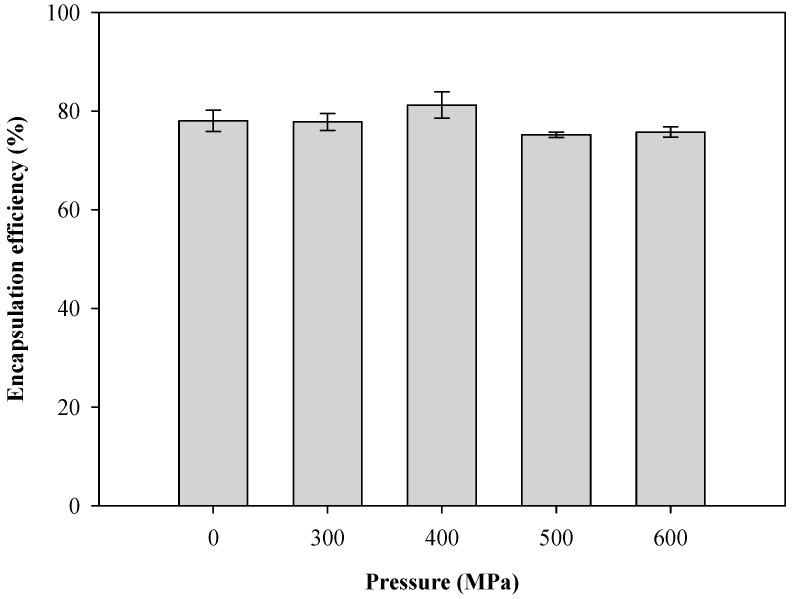
Effect of pressure on encapsulation efficiency of CTP liposomes prepared by the HPP method.

Liposomes are nano-scale spheroid vesicles composed of one or multiple lipid bilayer(s) enclosing an aqueous core. As bilayer structures, liposomes in aqueous solutions can encapsulate hydrophilic substances in the aqueous compartment, while hydrophobic substances can be accommodated in the lipid phase. The effects of hydrostatic pressure on the encapsulation efficiency of CTP liposomes by HPP are shown in Figure 5. The encapsulation efficiency of the unpressurized sample was 78.03 ± 2.14%. The homogenization, either with or without HPP, had no significant impact on the encapsulation efficiency of the CTP liposomes. However, HPP homogenization was found to effectively reduce the particle size of the liposomes and arrived at more than 75% encapsulation efficiency.

### 3.3. Effects of Different Homogenization Types on Mean Particle Size, Pdi, And Encapsulation Efficiency of Liposomes

The effects of four different homogenization methods on the mean particle size, PDI, and encapsulation efficiency of the CTP liposomes are shown in Table 1, respectively. The mean liposome size via magnet stirring, bath-sonicated, probe-sonicated, and HPP treatments at 300 MPa were 645.8 ± 75.2 nm, 290.0 ± 9.1 nm, 137.1 ± 13.4 nm, and 240.7 ± 5.8 nm, respectively. Both HPP and sonication processing were found to successfully reduce the particle size of the liposomes. Furthermore, the results were also consistent with the TEM images of liposomes under different homogenization types, as shown in Figure 2. In addition, the results indicated that the encapsulation efficiency of the liposomes under HPP treatment (77.8 ± 1.7%) was also significantly higher than that under the ultrasonic bath (37.5 ± 11.3) and probe ultrasonication (53.7 ± 8.1) treatments due to the lowest PDI and the unimodal distribution. Compared to the probe ultrasonication, ultrasonic bath, and magnet stirring methods, the liposomes prepared by HPP at 300 MPa had the lowest PDI of 0.37±0.031 and had the best uniformity of size to avoid the liposomal aggregation and maintain vesicles with a stable diameter. The study [22] demonstrates that the optimal choice of encapsulation technique was based on the preparation process, the stable bilayer characteristics, the suitable particle size, the desired PDI, and the physicochemical properties of functional compounds encapsulated in lipid. The research [23] also revealed that both the mean particle size and PDI of liposomes prepared by the probe sonication method were lower than those prepared by the bath sonication method, but the encapsulation efficiency was as much as twice as high for probe ultrasonication than the ultrasonic bath.

### 3.4. Mechanisms of Liposome Formation via the HPP Method

**Figure 6:**
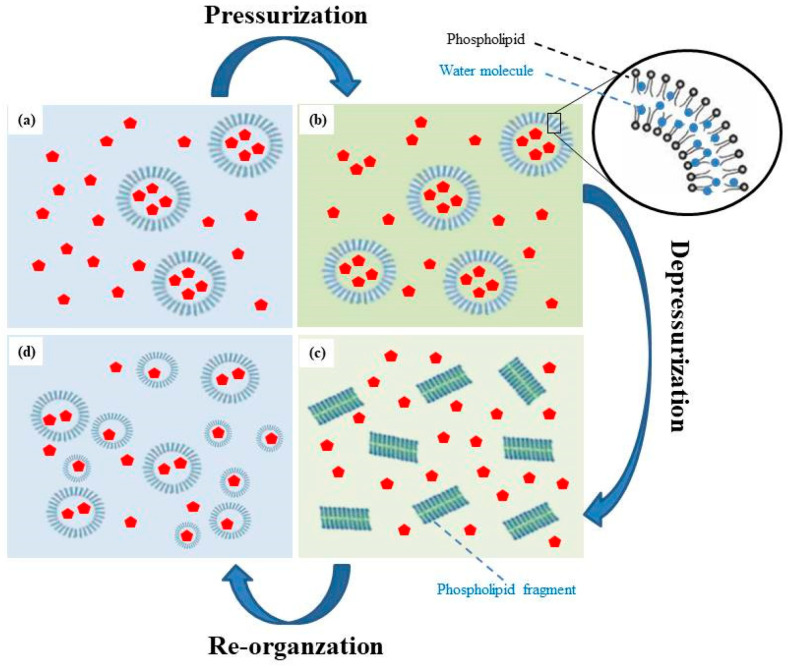
The mechanism of liposome formation by the HPP method (**a**) Injection ethanol and phospholipids; (**b**) In pressurization processing; (**c**) During decompression; (**d**) In re-organization processing.

The possible mechanism of liposome formation via the HPP method is illustrated in Figure 6. In summary, CTP liposomes were formed by a pressurization and depressurization process, which produced a dispersion and destruction effect via the fast-adiabatic expansion of water. The mechanism consisted of four phases:

1. When ethanol and dissolved phospholipids are injected into an aqueous buffer, the spontaneous formation of liposomes was observed.
2. In pressurization processing, large molecules such as phospholipids were compressed by hydrostatic pressure, which may cause small molecules such as water molecules to penetrate and fill the spaces between phospholipid molecules. Simultaneously, the double-layered phospholipids of the external membrane were packed tightly in the compression stage, thus promoting the transition to a gel state. Random movements of the phospholipid acyl chains produced by HPP caused water molecules to infiltrate between the hydrophilic phospholipid head groups and into the bilayers [24,25].
3. During decompression, the structure of the phospholipid bilayer was lost and pores were formed due to the fast adiabatic expansion of water. Water molecules rapidly escaped from the pores and break up the structure of the phospholipid bilayer to form transient phospholipid fragments. In microbial cells and spores, fast decompressions may lead to higher inactivation due to the fast-adiabatic expansion of water [25,26]. The previous studies proposed that the aqueous phase was first mixed with the phospholipid, supercritical fluid, and co-solvent mixture and later rapidly decompressed by spraying through a nozzle [25]. The size of liposomes obtained was in the range of 0.2–4 m [27].
4. After the formation of the transient solution, the temporarily separated phospholipids and cholesterol recombined rapidly due to the Van Der Waals force and hydrophobic interactions. In re-organization processing, these fragments self-assembled in solutions to form liposomes with a smaller particle size and a uniform distribution.

## 4. Conclusions

The microencapsulation of CTP extracts in liposomes was successfully achieved without co-solvents at room temperature within 10 min using the eco-friendly HPP technology. Based on the optimal hydrostatic pressure of 300 MPa in the pressurization and depressurization processes, the encapsulation efficiency of the CTP extracts was determined to be 77.8% in the liposomes, with a mean particle size of 240.7 nm and a PDI of 0.37. The mean particle size of the liposomes could be effectively reduced by HPP treatment. The PDI and encapsulation efficiency of liposomes, prepared using HPP, were superior to those in conventional methods (probe ultrasonication, ultrasonic bath, and magnet stirring) of liposome preparation. A possible mechanism of CTP liposome formation was proposed based on the experimental results, which indicated that the formation of CTP liposomes with a small particle size, low PDI, and high encapsulation efficiency was due to the appropriated hydrostatic pressure via the fast-adiabatic expansion of water, which plays an important role in HPP technology and could prevent liposome particles from agglomerating with each other. HPP using a high pressure-packed tower can not only affect their size and distribution, leading to the production of liposomes, but also be used to operate non-thermal processing for the prevention of thermal-sensitive substance. The HPP method developed in this study could offer a large-scale, sustainable, and energy-saving technique for the rapid production of integrated liposomes with a unique capability to microencapsulate bioactive compounds of thermal degradation for cosmetic and pharmaceutical applications.

## Figures and Tables

**Table 1:** Effects of different homogenization types on mean particle size, PDI, and encapsulation efficiency of CTP liposomes.

Homogenization Type	Mean Particle Size (nm)	PDI	Encapsulation Efficiency (%)
Magnet stirring	645.8 ± 75.2	0.88 ± 0.10	78.0 ± 2.1
Ultrasonic bath	290.0 ± 9.1	0.47 ± 0.08	37.5 ± 11.3
Probe ultrasonication	137.1 ± 13.4	0.44 ± 0.07	53.7 ± 8.1
HPP at 300 MPa	240.7 ± 5.8	0.37 ± 0.03	77.8 ± 1.7

## Data Availability

The data presented in this study are available in [A New Approach for the Microencapsulation of Clitoria Ternatea Petal Extracts by a High-Pressure Processing Method].
